# Quality of cardiopulmonary resuscitation in out-of-hospital cardiac arrest before and after introduction of a mechanical chest compression device, LUCAS-2; a prospective, observational study

**DOI:** 10.1186/s13049-015-0114-2

**Published:** 2015-04-22

**Authors:** Tinne Tranberg, Jens F Lassen, Anne K Kaltoft, Troels M Hansen, Carsten Stengaard, Lars Knudsen, Sven Trautner, Christian J Terkelsen

**Affiliations:** Department of Cardiology, Aarhus University Hospital, Aarhus, Denmark; Department of Prehospital Medical Care Service, Central Denmark Region, Aarhus, Denmark; Prehospital Critical Care Team, Aarhus University Hospital, Aarhus, Denmark; Helicopter Emergency Medical Service, Central Denmark Region, Aarhus, Denmark; Falck A/S, Copenhagen, Denmark

**Keywords:** Out-of-hospital cardiac arrest (OHCA), Resuscitation, CPR quality, Mechanical chest compression

## Abstract

**Background:**

Mechanical chest compressions have been proposed to provide high-quality cardiopulmonary resuscitation (CPR), but despite the growing use of mechanical chest compression devices, only few studies have addressed their impact on CPR quality. This study aims to evaluate mechanical chest compressions provided by LUCAS-2 (Lund University Cardiac Assist System) compared with manual chest compression in a cohort of out-of-hospital cardiac arrest (OHCA) cases.

**Methods:**

In this prospective study conducted in the Central Denmark Region, Denmark, the emergency medical service attempted resuscitation and reported data on 696 non-traumatic OHCA patients between April 2011 and February 2013. Of these, 155 were treated with LUCAS CPR after an episode with manual CPR. The CPR quality was evaluated using transthoracic impedance measurements collected from the LIFEPAK 12 defibrillator, and the effect was assessed in terms of chest compression rate, no-flow time and no-flow fraction; the fraction of time during resuscitation in which the patient is without spontaneous circulation receiving no chest compression.

**Results:**

The median total episode duration was 21 minutes, and the episode with LUCAS CPR was significantly longer than the manual CPR episode, 13 minutes vs. 5 minutes, p < 0.001. The no-flow fraction was significantly lower during LUCAS CPR (16%) than during manual CPR (35%); difference 19% (95% CI: 16% to 21%; p < 0.001). No differences were found in pre- and post-shock no-flow time throughout manual CPR and LUCAS CPR.

Contrary to the manual CPR, the average compression rate during LUCAS CPR was in conformity with the current Guidelines for Resuscitation, 102/minute vs. 124/minute, p < 0.001.

**Conclusion:**

Mechanical chest compressions provided by the LUCAS device improve CPR quality by significantly reducing the NFF and by improving the quality of chest compression compared with manual CPR during OHCA resuscitation. However, data on end-tidal Co_2_ and chest compression depth surrogate parameters of CPR quality could not be reported.

## Introduction

High-quality chest compression and early defibrillation are particularly essential for survival outcome after out-of-hospital cardiac arrest (OHCA) [1]. Recent guidelines for resuscitation underline the importance of quality chest compressions and of minimising the time with no chest compressions during cardiopulmonary resuscitation (CPR) [2]. However, studies have shown that chest compressions performed by health care professionals do not meet the recommendations for compression rate, depth and continuity, which results in considerably longer no-flow times than necessary and desirable [3,4].

**Figure 1:**
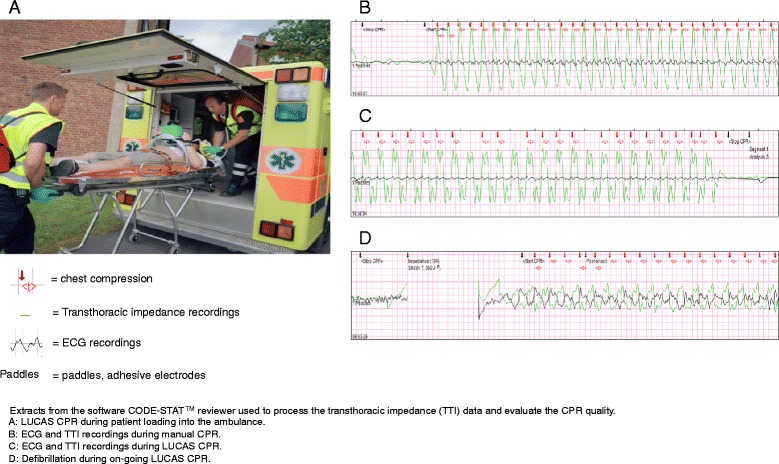
LUCAS and extracts from the software CODE-STAT reviewer used to process the transthoracic impedance (TTI) data and evaluate the CPR quality. **A**: LUCAS CPR during patient loading into the ambulance. **B**: ECG and TTI recordings during manual CPR. **C**: ECG and TTI recordings during LUCAS CPR. **D**: Defibrillation during on-going LUCAS CPR.

To address these shortcomings, a mechanical chest compression device, LUCAS-2 (Lund University Cardiopulmonary Assist System), has been developed (Figure 1A). The LUCAS device has been proposed to provide high-quality chest compressions whereby the interruptions seen with manual CPR [5] may be avoided; and randomised animal studies have demonstrated significantly better coronary perfusion pressure and cerebral artery blood flow when LUCAS-2 is used than when manual CPR is performed [6].

Despite the growing use of these devices, randomised studies in humans have not been able to show better outcome for OHCA patients resuscitated with mechanical chest compressions [7-9]. Research comparing the effect of manual CPR and mechanical CPR in the same patient is limited. The present study aimed to implement LUCAS in the physician-manned prehospital critical care teams and the helicopter emergency medical service (HEMS) and to evaluate if the mechanical chest compressions provided by LUCAS improve CPR quality compared with manual chest compressions in OHCA patients.

## Patients and methods

### Setting

The emergency medical service (EMS) in the Central Denmark Region is organised as a two-tier system; it operates a double-dispatch service that covers an area of 13,142 km^2^ inhabited by a total of 1.3 million people. The first tier consists of 75 conventional ambulances manned with two EMS providers. The second tier consists of nine physician-manned prehospital critical care teams and one HEMS available 24/7. The prehospital critical care teams and the HEMS have the competency to provide advanced life support (ALS). ALS includes the potential use of the LUCAS device. Conventional ambulances provide basic life support (BLS) and defibrillation only.

The EMS in the Central Region Denmark has a standardised pre-hospital-resuscitation protocol, which was strictly adhered to in this study. A conventional ambulance is dispatched to all emergencies including OHCA. It normally arrives at the scene as a first responder. The prehospital critical care team or the HEMS is dispatched to patients with presumed OHCA as determined by the dispatcher triage and according to availability. All cardiac arrest patients are treated according to the 2010 Guidelines for Resuscitation. During the entire study period, the physicians of the prehospital critical care team and the HEMS serve jointly as the decision-maker who decides whether or not the LUCAS device is to be used in the attempt of resuscitation. In patients receiving LUCAS CPR, the EMS providers initiate the BLS until the arrival of the physician-manned mobile prehospital critical care team bringing the LUCAS device. Manual CPR is continued when the LUCAS device is being deployed; and in order to minimise the interruptions in CPR, it is only paused briefly when the back plate is inserted and the arms positioned. When the prehospital critical care team or the HEMS are involved, the patient is transported directly to the tertiary university hospital’s Heart Centre, which is staffed with dedicated anaesthesiologists, cardiologists and surgeons. Provided that active treatment is indicated, acute coronary angiography (CAG) and/or percutaneous coronary intervention (PCI) is performed, and therapeutic hypothermia is applied according to international guidelines. The patients are subsequently treated in the Cardiac Intensive Care Unit which is staffed by anaesthesiologists and cardiologists. However, if the prehospital critical care team or the HEMS are unable to assist the EMS providers, the operating procedure for the EMS providers state that after three defibrillation attempts or in the case of a non-shockable rhythm after three 2-minute cycles of CPR and persisting cardiac arrest, the patient is transported to the nearest hospital.

A total of nine LUCAS devices were introduced in the prehospital critical care team and the HEMS. Prior to this introduction, the physicians and the EMS providers were informed about the study protocol and the procedures, and they were instructed how to handle the LUCAS device. Furthermore, they accomplished a manikin-scenario-training-session, which enabled them to use the device correctly, i.e. to deploy the device within 20 seconds while minimising interruptions in CPR. The physicians and the EMS providers who did not have the opportunity to attend the training session were trained and accredited by one of the physicians who took part in the session.

### Study design

The inclusion criteria for the present study were non-traumatic OHCA, age above 18 years and OHCA occurring between 1 October 2011 and 31 January 2013. Exclusion criteria were pregnancy, trauma, intoxication, inability to attach the LUCAS device to the patient, patients with missing data and total resuscitation episode < 2 minutes.

**Figure 2:**
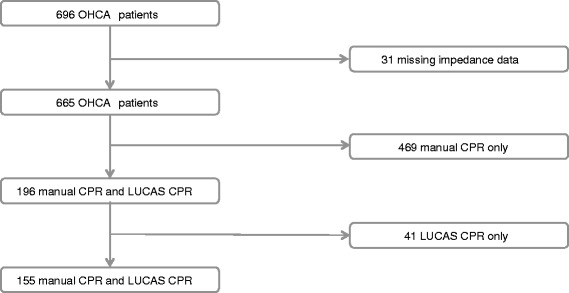
Study population.

Data were collected prospectively and registered according to the Utstein templates for resuscitation registries [10] (Figure 2). The Danish Data Protection Agency (file number: 2013-41-1758) and The National Board of Health approved the present study, and the Regional Ethics Committee concluded that no formal approval was necessary because the study was designed as a quality-control study.

### Data collection

Standard LIFEPAK 12 defibrillators (Physio-Control, Redmond, WA, USA) were used. De defibrillators continuously measure the transthoracic impedance (TTI) by applying a near constant current across the defibrillations pads. After a CPR effort, the ECGs and the TTI data of the OHCA case were transferred to a central server at the tertiary hospital; Aarhus University Hospital, Denmark. Furthermore, the prehospital critical care teams and the HEMS filled out a separate study form regarding the end-tidal CO_2_ (ETCO_2_), which served as a surrogate marker of CPR quality.

The software programme CODE-STAT™ -8 (Physio-Control Inc., Redmond, WA) was used to process the TTI data (Figure 1B, C, D). The software automatically annotates the chest compressions; however, each OHCA case was verified at the level of single compressions. Any incorrect automatic annotations were deleted, new annotations were added when annotations were missing; and the system calculated pre-shock and post-shock pauses, the compression rate and the actual number of compressions per minute, no-flow time (NFT) and no-flow fraction (NFF). The NFT, defined as the time without return of spontaneous circulation (ROSC) and the time without chest compressions, is a validated measure of the CPR quality, and it is reported according to previously published definitions [11-13]. The term NFF is defined as NFF = (NFT/episode duration – time with ROSC). The NFF represents the proportion of interruptions in CPR during the episode. The actual number of chest compressions delivered per minute represents both the compression rate and the pauses in the compressions.

We defined the CPR-pause interval as the time from the trailing edge of the last chest compression to the leading edge of the next chest compression. Mechanical compressions were distinguished from the manual compressions by their highly regular morphology (Figure 1C). Usable data files included TTI data for the entire episode, from the first manual chest compression to the end of the final mechanical chest compression. The case was excluded if the total episode was shorter than 2 minutes.

Additionally, nationally adapted Utstein style forms filled out by the EMS personnel were used to obtain data on bystander CPR, OHCA location and the use of an automated external defibrillator (AED) prior to arrival of the EMS and whether OHCA was witnessed or unwitnessed. Data on the patients who achieved ROSC, survived to hospital discharge and survived after 30 days were collected from the OHCA registration form, ambulance records and hospital records.

### The properties of LUCAS-2

LUCAS is a chest compression system that provides both active compression and decompression of the chest wall back to neutral position (Figure 1A). The first generation of the device, LUCAS-1, was driven by compressed air which was superseded by a battery-driven device in 2009. The device consists of a silicone rubber suction cup that is applied to the patient’s chest and a cylinder mounted on two legs connected to a stiff back palate. It delivers compressions at a rate of 102 per minute and at a depth between 5 and 6 cm as prescribed in the guidelines for resuscitation provided by The European Resuscitation Council (ERC).

### Statistics

Data management and statistical calculations were performed using STATA/SE 12.1. Normally distributed continuous variables are presented as mean ± standard deviation and non-normally distributed variables as median and quartiles. Differences were analysed with Student’s paired t-test or Wilcoxon’s sign rank test as appropriate. Categorical variables are presented as number (n) and percent (%) as appropriate, and differences are analysed with McNemar’s test. The a priori α-level was 0.05.

## Results

Data were reported on 696 OHCA patients who were attempted resuscitated between 1 October 2011 and 31 January 2013. Of these, 196 were resuscitated with both manual CPR and LUCAS CPR. Due to incomplete TTI data, 31 (4%) patients were excluded. Another 41 (21%) patients were excluded due to the fact that only data with LUCAS CPR were available. The remaining 155 OHCA patients comprised the study population (Figure 2).

Table 1 shows the baseline characteristics of the study population. The mean age was 66 years (SD = 15). OHCA occurred particularly in men (67%) at home (81%) who presented a non-shockable rhythm at first rhythm analysis (64%). OHCA was witnessed by either laypersons, health care professionals or EMS in 67% (83/124) of the cases. Bystander CPR was provided in 74% (93/125) of the cases. None of the patients in the study population were treated with a private or public AED before arrival of the EMS. The median time (interquartile range, IQR) from emergency call to EMS arrival was 5 (3–9) minutes, and the median time (IQR) from emergency call to first rhythm analysis was 9 (7–14) minutes.

**Table 1:** **Baseline characteristics, n = 155**

**Cardiac arrest background variables**	
Age in years, mean (SD)	66 (±15), n = 146
Male gender 67%	(92/138)
Place of cardiac arrest	81% (99/123)
Home	19% (23/123)
Public	1% (1/123)
EMS vehicle	81% (99/123)
Cardiac arrest witnessed	67% (83/124)
Layperson	53% (66/124)
Health care professional	5% (6/124)
EMS	9% (11/124)
Bystander CPR	74% (93/125)
Rhythm on arrival of EMS	
Asystole	40% (50/126)
PEA	24% (31/126)
VF/VT	33% (41/126)
Other	3% (4/126)
Proportion of AED analysis	98% (152/155)
+ defibrillation	47% (73/155)
- defibrillation	87% (135/155)
Defibrillation with AED before arrival of the EMS	0% (0/155)

Continuous data presented as mean +/− SD, valid cases.

Categorical variables presented as percentage (n/valid cases).

EMS = Emergency medical service. Health care professional = EMS personnel, nurse, physician. CPR = cardiopulmonary resuscitation. PEA = Pulseless electrical activity. VF = Ventricular fibrillation. VT = Ventricular tachycardia. AED = Automated external defibrillator.

Among the patients with a shockable rhythm as the presenting heart rhythm, the time (IQR) from emergency call to first defibrillation by the EMS was 9 (8–19) minutes.

CPR variables are shown in Tables 2 and 3. The mean NFF was significantly lower during the LUCAS episode ((16%; 95% CI: 15; 18) than during the manual CPR episode (35%; 95% CI: 33; 37) (p < 0.001)). In addition, the chest compression rate and the actual number of compressions per minute were significantly lower throughout the LUCAS episode than throughout the manual CPR episode.

**Table 2:** **Quality of cardiopulmonary resuscitation (CPR)**

**CPR variables**	**Manual CPR**	**LUCAS CPR**	**P-value**
	**n = 155**	**n = 155**	
Episode duration, min.	5 (2; 6)	13 (11; 14)	<0.001
No-flow fraction, %	35 (33; 37)	16 (15; 18)	<0.001
Chest compression rate per minute	124 (121; 126)	102 (102; 102)	<0.001
Number of chest compressions per minute	75 (72; 79)	84 (82; 85)	<0.001

No-flow time = Time without ROSC – time without chest compressions.

No-flow fraction = No-flow time/(episode duration – time with ROSC). ROSC = Return of spontaneous circulation.

CPR variables are presented as mean values (95% CI).

**Table 3:** **No-flow time (NFT) and rhythm analysis with/without defibrillation**

**CPR variables**	**Manual CPR**		**LUCAS CPR**		
	**Mean (95% CI) sec.**	**Number with data**	**Mean (95% CI) sec.**	**Number with data**	**P-value**
NFT in relation to AED analysis	15 (13; 18)	60	16 (13; 19)	55	0.620
+ defibrillation					
NFT in relation to AED analysis	17 (16; 18)	112	18 (16; 20)	101	0.960
- defibrillation					
Pre-shock NFT	17 (15; 20)	60	20 (16; 23)	55	0.406
Post-shock NFT	7 (6; 8)	60	7 (6; 9)	55	0.466

No-flow time (NFT) = Time without ROSC – time without chest compressions. ROSC = Return of spontaneous circulation. AED = Automated external defibrillator.

There was no significant difference between the results of either NFT during rhythm analysis with and without defibrillation or pre- and post-shock NFT with manual or LUCAS CPR (Table 3). LUCAS NFT was median (IQR = 24 (14–38)) seconds.

Table 4 presents survival rates and treatment of hospitalised OHCA patients. Forty-five patients (29%) were admitted to hospital alive and 14 (9%) were discharged alive. Acute CAG was performed in 31 (60%) of the patients. In six cases, the angiography was performed while LUCAS CPR was still being performed. Eight patients (15%) underwent primary PCI, and therapeutic hypothermia was induced in 27 (52%) patients. Two (4%) patients were treated with cardiopulmonary support. Both were admitted to hospital with on-going LUCAS CPR and both were alive after 30 days. In the remaining 103 (66%) patients in whom CPR was deemed futile, treatment was terminated on the scene or upon admission to hospital. We found no differences in age or comorbidity among survivors and non-survivors.

**Table 4:** **In-hospital treatment among patients admitted alive (n = 45) and among patients admitted with on-going LUCAS CPR (n = 7), n = 52**

**Treatment**	**Admitted alive (n = 45)**	**Ongoing LUCAS CPR (n = 7)**	**Total (n = 52)**
Coronary angiography	56% (25)	86% (6)	60% (31)
Percutaneous coronary intervention	16% (7)	14% (1)	15% (8)
Therapeutic hypothermia	58% (26)	14% (1)	52% (27)
Cardiopulmonary support	0% (0)	29% (2)	4% (2)

Categorical variables presented as percentage (valid cases).

## Discussion

While recent years have seen studies reporting on the outcome associated with mechanical CPR [7,14], only few studies have provided data on the quality of mechanical chest compressions, and those that have are limited by a low number of patients [15]. The present study evaluates the performance and quality of both manual and mechanical CPR in the same patient primarily based on TTI.

Our results show that during the manual episode, NFF lasted 34% of the time in patients without ROSC. However, once the LUCAS device was deployed, the NFF was reduced significantly to 16% of the time. Although an NFF of 34% during manual CPR is lower than what has been reported in previous studies where CPR was performed according to the former resuscitation guidelines of 2005 [4], we still find that the NFF is too high. Its size stresses the importance of short pauses during resuscitation and the importance of offering CPR training with performance feedback to further improve CPR quality. The average NFF of only 16% achieved with the LUCAS device was low. One could speculate that rhythm analysis was not performed every second minute as prescribed by the current guidelines. However, we have no data to substantiate this because the CODE-STAT does not allow registrations of the manual rhythm analysis. Thus, ambulance personnel have to use the automatic defibrillator mode as documented in the data derived from the defibrillator; inversely, physicians normally interpret the rhythm themselves to reduce CPR interruptions.

The low NFF with the LUCAS device may also have been achieved owing to fewer interruptions while loading the patient into the ambulance and during transport with on-going LUCAS.

Another advantage of the LUCAS device is that it affords the possibility of delivering shocks during compression and enables users to shorten or eliminate pauses for defibrillation. Nevertheless, we found no difference in pre-shock and post-shock NFT in relation to automated rhythm analysis throughout the time with manual CPR or LUCAS CPR. The median pre-shock pause was 17 sec. (IQR 15–20) and the median post-shock pause was 7 sec. (IQR 6–8). While these pauses are reasonable for defibrillation in automatic mode during manual CPR and similar to pauses reported in previous research [12], they are suboptimal for defibrillation during LUCAS CPR. However, the TTI analysis of all cases at the level of single chest compression revealed that the pre-shock and the post-shock NFT during LUCAS CPR might be overestimated due to the inability of CODE-STAT to register chest compressions when shock is delivered. The chest compression rate and the actual number of compressions per minute, which are important elements in CPR quality, were significantly better performed during LUCAS CPR. The chest compressions were delivered at a rate higher than recommended, and they were probably also too shallow during manual CPR (124 compressions per minute), which may result in failure to achieve ROSC.

Although they fell short of being optimal, the manual chest compression rate and the number of chest compressions per minute still appear superior to the manual compression rates (138 compressions per minute) reported in the study by Krarup et al. [4].

The episode duration with LUCAS CPR was nearly three times longer than the manual CPR episode (Table 3). This agrees well with previous reports, and it presumably reflects that these cases are the ones with the longest resuscitation attempt [16].

In a study of changes in bystander resuscitation attempts and survival during a 10-year period in which national initiatives were taken to improve rates of bystander CPR, Wissenberg et al. [17] concluded that an increase in survival following OHCA was associated with an increase in bystander CPR, regardless of witnessed cardiac arrest. This is consistent with the findings in our study, which showed a large proportion of patients with cardiac arrest at home and a high rate of bystander CPR. Our study was not designed or statistically powered to evaluate survival outcome. Nevertheless, the 30-day survival after OHCA resembles the previously described values of 9% [17,18]. This was achieved despite the fact that the present cohort may consist of a selected high-risk group in need of prolonged CPR as compared with patients who are easily resuscitated within a few minutes after start of CPR or defibrillation. The two patients admitted to hospital with on-going LUCAS CPR and who were treated with cardiopulmonary support were resuscitated during a particularly long time; regardless of this, both patients were alive after 30 days with minimal neurological sequelae. We believe that it is unlikely that these patients would have survived if transported and treated with manual CPR only. Furthermore, manual CPR is cumbersome during catheterisation and potentially hazardous for the health care personnel due to radiation exposure. Therefore, the LUCAS device seems ideal during catheterisation compared with manual CPR [19].

### Limitations

There are some limitations to this study. First, it is a small observational, prospective study with potentially unknown confounders; however, the relative advantage of this study design is that being their own controls, the patients studied are the same during manual CPR and LUCAS CPR. Second, the physician of the prehospital critical care team decides if the LUCAS device is to be used in the resuscitation, which undoubtedly introduces selection bias. Third, even though TTI and ECG data transfer to the central server was mandatory, a large part of the OHCA data was never transferred to the server.

Ventilation and compression depth measurements during resuscitation are recommended for the evaluation of CPR quality; however, these measurements cannot be assessed from TTI data alone, and little data support the clinical importance of these variables in this setting [13]. The prehospital critical care teams were to take part in filling out a separate study form regarding the end-tidal CO_2_ (ETCO_2_), which is considered a surrogate measure of cardiac output during CPR [20]; however, little evidence exists to support this concept. Unfortunately, we are not able to report the ETCO_2_ values in this study due to the lack of registered values/too many missing values. Data on adverse device events or injuries have likewise not been reported in those patients not surviving OHCA due to the fact that these events were not described in the medical reports. The 45 patients admitted to hospital alive did not have any device-related events or injuries.

## Conclusion

Mechanical chest compressions provided by the LUCAS device improve CPR quality by significantly reducing the NFF and by improving the quality of chest compression compared with manual CPR during OHCA resuscitation.

### Perspectives

Randomised clinical studies not have been able to demonstrate improved survival for patients resuscitated with mechanical chest compression devices, and the devices have to some extent been discredited following the ASPIRE trial, the LINC trial and the PARAMEDIC trial. However, these trials are impeded by methodological deficiencies such as different resuscitation protocols in the compared groups and, in particular, prolonged time to deployment of the device. Maintenance of high-quality CPR during OHCA is not easy because of the small number of crew present, fatigue, patient access, and the impossibility of performing resuscitation in a moving vehicle. These considerations must be balanced against the current evidence from previous studies when deciding on the future role of mechanical CPR in healthcare systems, but it seems reasonably that such devices will continue to play a role when manual CPR is impractical. Nevertheless, it is essential that resources are available to support regular training of healthcare personnel ensuring that resuscitation guidelines are followed and to monitor CPR quality continuously.
